# Relationship between heart rate recovery and inflammatory markers in patients with polycystic ovary syndrome: a cross-sectional study

**DOI:** 10.1186/1757-2215-2-3

**Published:** 2009-02-02

**Authors:** Francesco Giallauria, Francesco Orio, Gaetano Lombardi, Annamaria Colao, Carlo Vigorito, Maria Giovanna Tafuri, Stefano Palomba

**Affiliations:** 1 Department of Clinical Medicine, Cardiovascular and Immunological Sciences, Cardiac Rehabilitation Unit, University of Naples "Federico II", Naples, Italy; 2 Endocrinology, Faculty of Exercise Sciences, University of Naples "Parthenope", Naples, Italy; 3 Department of Molecular & Clinical Endocrinology and Oncology, University of Naples "Federico II", Naples, Italy; 4 Teaching and Methods of Sportive Activity, Faculty of Exercise Sciences, University of Naples "Parthenope", Naples, Italy; 5 Department of Obstetrics and Gynaecology, University of Catanzaro "Magna Graecia", Catanzaro, Italy

## Abstract

**Background:**

Polycystic ovary syndrome (PCOS) is an endocrine disease closely related to several risk factors for cardiovascular disease. An abnormal heart rate recovery (HRR), an easily-obtained measure derived from exercise stress test and closely related to an increased risk for cardiovascular mortality, has been recently described in PCOS women. A subclinical increase of the inflammation markers has been also observed in the PCOS. This study was designed to study the relationships between HRR and inflammatory markers in PCOS women.

**Methods:**

Two-hundred forty-three young PCOS patients without known risk factors for cardiovascular risk were enrolled. All patients underwent hormonal and metabolic profile, white blood cells (WBCs) count and C-reactive protein (CRP). HRR was calculated as the difference between heart rate at peak exercise and heart rate at first minute of the cool-down period. Abnormal HRR was defined as ≤18 beats/min for standard exercise testing.

**Results:**

Eighty-nine out of 243 patients presented abnormal HRR. Serum CRP (1.8 ± 0.7 vs. 1.1 ± 0.4 mg/dl, *p *< 0.001) and WBCs (7.3 ± 1.8 vs. 6.6 ± 1.5 10^9 ^cells/l, *p *< 0.001) concentrations were significantly higher in PCOS patients with abnormal *versus *normal HRR. HRR was significantly associated with both CRP (r = -0.33, *p *< 0.001) and WBCs (r = -0.29, *p *< 0.001), although in a stepwise multiple regression HRR resulted independently associated with CRP (beta = -0.151, p = 0.001) alone. In a logistic multivariate model, the group within the highest quartile of CRP (odds ratio 1.59, 95% CI 1.07–2.33) was more likely to have abnormal HRR than those within the lowest quartile.

**Conclusion:**

Abnormal HRR and inflammatory markers are closely associated in PCOS women acting probably in concert to increase the cardiovascular risk profile of these patients.

## Background

Mounting evidences suggest that polycystic ovary syndrome (PCOS) is a complex endocrine-metabolic disease strictly associated with long-term cardiovascular risk [1,2]. In fact, PCOS women, even at young age, have a clustering of cardiovascular risk factors [3-7] suggesting that they represent a group with an increased risk for developing early-onset cardiovascular disease [2,8].

Heart rate recovery (HRR) is an easily-obtained measure derived from exercise stress test and is defined as the fall in heart rate during the first minute after maximal exercise [9]. The underlying mechanisms by which slow HRR after exercise is associated to an increased risk for cardiovascular mortality [10] are not fully understood. Because the rapid decrease in heart rate immediately after exercise is primarily due to vagal reactivation [11-13], slow HRR may be indicative of decreased autonomic nervous system activity.

In a recent study, PCOS women showed slower HRR when compared to healthy controls [14]. Notably, slower HRR was inversely associated to body mass index (BMI) and to markers of insulin resistance, suggesting a close and complex relationship between autonomic function and glucose metabolism in PCOS women [2,14].

Inflammation plays a key role in the pathophysiological mechanism of atherosclerosis [15,16] and cardiovascular disease [17]. Several inflammation markers, such as C-reactive protein (CRP), interleukin 6, soluble intercellular adhesion molecule type 1, and white blood cells (WBCs) count, are found to be significant predictors of the risk of coronary heart disease and future cardiovascular events [18]. Inflammation may also be associated with the metabolic syndrome [19,20] and increases WBCs count [21].

Cross-sectional studies have suggested that cardiac autonomic nervous activity, as assessed by heart rate variability, is related to inflammatory markers such as CRP and WBCs count [22,23]. Experimental studies reported that vagal nerve stimulation can modulate inflammatory cytokines through the cholinergic anti-inflammatory pathway [24,25], thus suggesting that HRR may be related to inflammatory markers.

Based on these considerations, the current study was designed to study the relationships between HRR and inflammatory markers in a population of women with PCOS.

## Methods

### Study design

Two-hundred forty-three PCOS patients were recruited at the Department of Molecular and Clinical Endocrinology and Oncology in Naples (Italy) among those who consecutively visited the ambulatory from January 2005 to March 2008.

All PCOS patients achieved the European Society for Human Reproduction and Embryology/American Society for Reproductive Medicine criteria for the PCOS diagnosis [26]. Polycystic ovaries were identified by transvaginal ultrasonography examination [26] and hirsutism by Ferriman-Gallwey score > 8.

Exclusion criteria included pregnancy, glucose intolerance [as screened by a 2-hour oral glucose tolerance test (OGTT)] and diabetes, hypothyroidism, hyperprolactinemia, Cushing's syndrome, non-classical congenital adrenal hyperplasia, and use of oral contraceptives, glucocorticoids, antiandrogens, ovulation induction agents, antidiabetic, antipsychotic and antiobesity drugs or other hormonal drugs and antihypertensive within the previous 6 months. Subjects with neoplastic, hepatic, respiratory and any cardiovascular disorder or other concurrent medical illness (i.e. respiratory and heart failure and renal disease) were also excluded from the study.

The study was conducted according to the guidelines of the Declaration of Helsinki, and the Institutional Ethical committee approved the study protocol. The purpose of the protocol was explained to each subject, and written informed consent was obtained from each patient before the screening. The study had no external funding source.

### Biochemical Assays

All blood samples were obtained in the morning between 08.00 h and 09.00 h after an overnight fasting during the early follicular phase (2nd–4th day) of progesterone-induced menstrual cycle. Blood samples were collected into tubes containing EDTA after a 30-min resting period in the supine position. All blood samples were immediately centrifuged at 4°C for 20 min at 1600 g, and stored at -20°C until assayed.

Plasma LH, FSH, prolactin (PRL), estradiol (E2), P, 17α-hydroxyprogesterone (17-OH-P), T, androstenedione (Δ4), and DHEA-S levels were measured by specific radioimmunoassays (RIA) as previously described [3-6]. The levels of SHBG were measured using an IRMA [3-6], and the free androgen index (FAI) was calculated [T(nmol/l)/SHBG(nmol/l) × 100].

Blood insulin and glucose levels were measured by a solid-phase chemiluminescent enzyme immunoassay and the glucose oxidase method, respectively [3-6]. The glucose and insulin areas under curve (AUC) for glucose (AUC_GLU_) and for insulin (AUC_INS_), and the AUC_GLU_/AUC_INS _ratio [27], in response to the OGTT were also calculated.

The lipid profile consisted of serum total cholesterol (TC), high-density lipoprotein-cholesterol (HDL-C), low-density lipoprotein-cholesterol (LDL-C) and triglycerides (TG) levels as previously described [3-6]. WBCs count and CRP were measured as previously described [4,7]. Inter- and intra-assay coefficients of variation were < 5% for all blood variables.

### Cardiopulmonary exercise test and HRR evaluation

PCOS women underwent a symptom-limited CPX with Bruce treadmill protocol [6]. Heart rate (HR) and blood pressure (BP) at baseline and peak exercise, heart rate 1 minute into a walking cool-down period (1.7 mph at 0% grade), and treadmill speed and grade at peak exercise were recorded as previously reported [14].

HRR was calculated as the difference between heart rate at peak exercise and heart rate at first minute of the cool-down period.

Abnormal HRR, determined in our population by finding the maximum value for the log-rank chi-square test statistic for all possible cutoff points across percentiles, was defined as ≤18 beats/min [9].

Respiratory gas exchange measurements were obtained breath-by-breath with use of a computerized metabolic cart (Vmax 29C, Sensormedics, Yorba Linda, CA) as previously described [6].

### Statistics

Data are expressed as mean ± standard deviation for continuous variables and as counts and proportions for categorical variables. Two group comparisons were performed using independent Student's t tests for continuous variables and the chi-square test for categorical variables. CRP values were log transformed because of non-normal distribution. Pearson's correlations, stepwise multiple regression analysis, and analysis of variance were used to determine the relation between HRR and inflammatory markers. The selection of variables for entrance into the multivariate model was based on the univariate analysis. We tested potential collinearity among covariates using Pearson's correlations. After these evaluations, we selected the non-collinear variables for multivariable models: age, BMI, AUC_INS_, fasting glucose, TC, HDL-C, TG, HR_REST_, and VO_2peak_. Logistic regression was used to evaluate the association between abnormal HRR (yes or no) and quartiles of CRP and WBCs count (categorical data). Statistical significance was set at p < 0.05 for all data. Statistical analyses were performed using SPSS version 13.0 (SPSS, Inc., Chicago, IL).

## Results

Group comparisons according to abnormal HRR (≤18 beats/min) are listed in Table 1. Abnormal HRR was found in 89 patients of 243 (36.6%). Patients with abnormal HRR had significantly greater levels of CRP (*p *< 0.001), and WBCs (*p *< 0.001), but not TC and LDL-C, than subjects with normal HRR.

**Table 1:** Hormonal characteristics of the PCOS population according to HRR.

**Variables**	**Normal HRR (n = 154)** **(> 18 beats/min)**	**Abnormal HRR (n = 89)** **(≤18 beats/min)**	***P *value**
**Ferriman-Gallwey score**	11.9 ± 3.5	12.1 ± 3.4	0.678
**FSH (IU/liter)**	10.5 ± 1.7	10.1 ± 1.6	0.725
**LH (IU/liter)**	24.2 ± 3.3	23.5 ± 3.1	0.811
**PRL (ng/ml)**	10.5 ± 1.3	10.2 ± 1.1	0.797
**E2 (pmol/liter)**	120 ± 30.5	118 ± 26.1	0.502
**P (nmol/liter)**	1.2 ± 0.4	1.3 ± 0.6	0.630
**17-OHP (nmol/liter)**	1.6 ± 0.3	1.5 ± 0.4	0.809
**T (nmol/liter)**	2.3 ± 0.7	2.5 ± 0.5	0.649
**A (nmol/liter)**	5.1 ± 0.7	5.3 ± 0.9	0.702
**DHEAS (μmol/liter)**	4320 ± 465	4290 ± 441	0.488
**SHBG (nmol/liter)**	27 ± 6.2	29 ± 6.5	0.549
**FAI**	8.5 ± 3.4	8.6 ± 3.6	0.882

Abbreviations: A, Androstenedione; DHEAS, dehydroepiandrosterone sulphate; E2, Estradiol; FAI, free androgen index; FSH, follicle stimulating hormone; HRR, heart rate recovery; LH, luteinizing hormone; P, progesterone; PRL, prolactin; SHBG, Sex Hormone Binding Globulin; T, testosterone; 17-OHP, 17α-hydroxyprogesterone.

Using Pearson's correlations, HRR was significantly associated with age (r = -0.28, p < 0.001), BMI (r = -0.49, *p *< 0.001), AUC_INS _(r = -0.44, *p *< 0.001), HDL-C (r = 0.26, *p *< 0.001), TG (r = -0.28, *p *< 0.001), HR_REST _(r = -0.31, *p *< 0.001), VO_2peak _(r = 0.51, *p *< 0.001), logCRP (r = -0.33, *p *< 0.001) and WBCs (r = -0.29, *p *< 0.001). For anthropometrical, metabolic and cardiopulmonary profile of the PCOS population according to HRR, see Table 2.

**Table 2:** Anthropometrical, metabolic and cardiopulmonary profile of the PCOS population according to HRR.

**Variables**	**Normal HRR (n = 154)** **(> 18 beats/min)**	**Abnormal HRR (n = 89)** **(≤18 beats/min)**	***P *value**
**Age (years)**	20.7 ± 2.3	23.9 ± 1.9	< 0.001
**BMI (Kg/m2)**	27.3 ± 2.9	31.3 ± 3.1	< 0.001
**WHR**	0.84 ± 0.2	0.87 ± 0.1	< 0.001
**TC (mg/dl)**	153.3 ± 18.1	155.8 ± 16.6	0.442
**HDL-C (mg/dl)**	44.2 ± 7.3	40.9 ± 10.5	< 0.001
**LDL-C (mg/dl)**	89.5 ± 8.3	90.4 ± 7.6	0.593
**TG (mg/dl)**	114.6 ± 20.3	122 ± 22.4	< 0.001
**Fasting glucose**	93.0 ± 6.8	98.3 ± 7.1	< 0.001
**Fasting insulin**	20.1 ± 3.3	24.2 ± 3.7	< 0.001
**AUC_GLU_**	11950 ± 2122	12033 ± 2154	0.335
**AUC_INS_**	16520 ± 950	17480 ± 970	< 0.001
**AUC_GLU/INS_**	0.77 ± 0.2	0.73 ± 0.3	< 0.001
**WBCs count (× 10^9 ^cells/l)**	6.6 ± 1.5	7.3 ± 1.8	< 0.001
**CRP (mg/dl)**	0.11 (0.09–0.25)	0.21 (0.11–0.69)	< 0.001
**logCRP (mg/dl)**	1.1 ± 0.4	1.8 ± 0.7	< 0.001
**HR_REST _(beats/min)**	67.2 ± 2.5	72.1 ± 3.9	< 0.001
**VO_2peak _(ml/Kg/min)**	26.1 ± 3.3	22.5 ± 2.8	< 0.001

Abbreviations: AUC_GLU_, area under the curve for glucose; AUC_INS_, area under the curve for insulin; BMI, body mass index; CRP, C-reactive protein; HDL-C, high density lipoprotein cholesterol; HRR, heart rate recovery; HR_REST_, resting heart rate; LDL-C, low density lipoprotein cholesterol; TC, total cholesterol; TG, triglycerides; VO_2max_, maximal oxygen consumption; WBCs, white blood cells; WHR, waist to hip ratio.

In the stepwise multiple regression analysis, HRR (each 1 beat/min) was independently associated with logCRP (Table 3). Subjects were divided into quartiles according to HRR (quartile 1: < 18 beats/min; quartile 2: 18 to 24 beats/min; quartile 3: 25 to 30 beats/min; quartile 4: > 30 beats/min).

**Table 3:** Stepwise multiple regression analysis for log C-reactive protein.

**Variables**	**β Coefficient**	**SE**	***P *value**
HRR*	-0.151	0.001	0.001
Fasting glucose	0.039	0.001	0.005
AUC_INS_	-0.136	0.001	0.001
BMI	0.126	0.003	0.001
VO_2max_	-0.139	0.001	0.001
HDL-C	-0.177	0.001	0.001
TG	-0.141	0.001	0.001
HR_REST_	0.108	0.002	0.001

Regression analysis included age, BMI, TC, HDL-C, LDL-C, TG, fasting glucose, AUC_INS_, HR_REST_, HRR, and VO_2max _as covariates. Abbreviations: AUC_INS_, area under the curve for insulin; BMI, body mass index; HDL-C, high density lipoprotein cholesterol; HR_REST_, resting heart rate; HRR, heart rate recovery; TC, total cholesterol; TG, triglycerides; VO_2max_, maximal oxygen consumption. *Each 1 beat/min.

**Figure 1:**
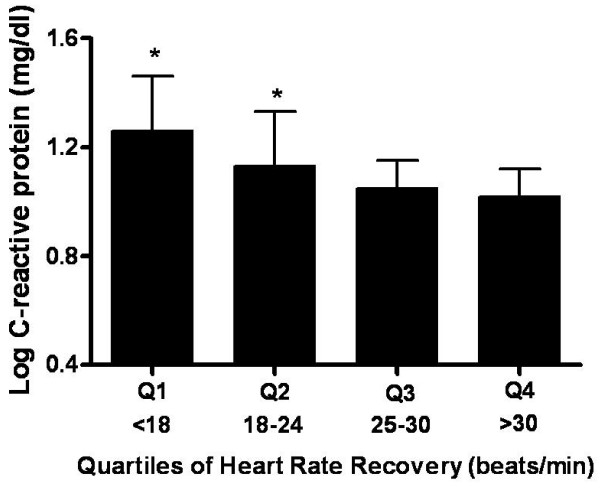
**Mean comparison of logCRP by quartile (Q) of HRR**. *Significantly different from Q4 (p < 0.05).

**Figure 2:**
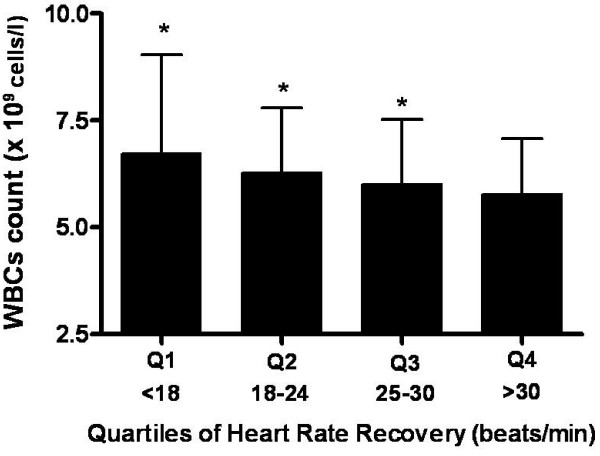
**Mean comparison of white blood cells (WBCs) count by quartile (Q) of heart rate recovery (HRR)**. *Significantly different from Q4 (p < 0.05).

The levels of logCRP and WBCs in each quartile were 1.26 ± 0.2, 1.13 ± 0.2, 1.05 ± 0.1, and 1.02 ± 0.1 mg/dl (Figure 1) and 6.74 ± 2.3, 6.29 ± 1.5, 6.02 ± 1.5, and 5.77 ± 1.3 × 10^9 ^cells/l (Figure 2) in quartiles 1 to 4, respectively. There were significant differences among quartiles 1 to 4 for logCRP (*p *< 0.05) and WBCs count (*p *< 0.05).

To evaluate the association between abnormal HRR and inflammatory markers, subjects were divided into quartiles according to logCRP and WBCs count. In a logistic multivariate model (adjusted for age, BMI, TC, HDL-C, TG, fasting glucose, AUC_INS_, HR_REST _and VO_2peak_) the group within the highest quartile of log CRP and WBCs count was more likely to have abnormal HRR than the group within the lowest quartile of log CRP and WBCs count (Table 4).

**Table 4:** Multivariate-adjusted odds ratios (95% confidence interval) for abnormal heart rate recovery (HRR) by quartile of log C-reactive protein (log CRP) and white blood cells (WBCs) count

**Variable**	**Quartile 1 (Reference)**	**Quartile 2**	**Quartile 3**	**Quartile 4**
**logCRP (mg/dl)**	(n = 67) ≤0.8	(n = 75) 0.81–1.19	(n = 53) 1.2–1.5	(n = 48) ≥1.5
Crude model	1.00	1.44 (1.21–1.86)	2.23 (1.73–2.88)	3.41 (2.42–4.25)
Adjusted model	1.00	1.19 (0.81–1.63)	1.34 (0.93–1.99)	1.59 (1.07–2.33)
**WBCs count (× 109 cells/l)**	(n = 62) ≤5.0	(n = 71) 5.1–6.0	(n = 58) 6.1–7.0	(n = 52) ≥7.1
Crude model	1.00	1.41 (1.14–1.93)	1.95 (1.48–2.65)	2.77 (2.19–3.58)
Adjusted model	1.00	1.38 (1.06–2.12)	1.33 (0.97–1.96)	1.61 (1.14–2.46)

Crude model is unadjusted. Adjusted model includes age, BMI, TC, HDL-C, TG, fasting glucose, AUC_INS_, HR_REST_, and VO_2peak_.

Abbreviations: AUC_INS_, area under the curve for insulin; BMI, body mass index; CRP, C-reactive protein; HDL-C, high density lipoprotein cholesterol; logCRP, logarithmic transformation of CRP values; HR_REST_, resting heart rate; TC, total cholesterol; TG, triglycerides; VO_2max_, maximal oxygen consumption; WBCs, white blood cells.

## Discussion

This study describes a significant association between post exercise slow HRR and increased levels of inflammatory markers in young PCOS women.

Post exercise HRR has been demonstrated as a risk factor for cardiovascular and all-cause mortality in healthy adults [10,28,29], in individuals with CVD [30] in individuals with risk factors for CVD [31,32] and in men with diabetes [33]. HRR is positively associated with insulin sensitivity as measured with a hyperinsulinemic-euglycemic clamp and metabolic syndrome in elderly men [34] as well as in middle-aged men and women [35-38].

HRR has also been found to be associated with individual components of the metabolic syndrome, such as blood glucose [36] low HDL-C [34,35] and resting systolic and diastolic blood pressure [35]. Panzer *et al. *[36] demonstrated a strong inverse relationship between fasting plasma glucose and HRR even at non-diabetic levels among middle-aged healthy men and women. Moreover, HRR has been shown to be inversely associated with triglyceride/HDL-C ratio in middle-aged healthy men and women [37].

Although IR is not a key criterion to diagnose PCOS [26], there is a wide consensus that subjects with PCOS are more insulin resistant than healthy women. IR has been found to be significantly associated with impaired cardiopulmonary functional capacity [6,7,38]. Moreover, abnormal HRR was significantly associated to BMI and to AUC_INS _(a powerful marker of IR), suggesting that impaired glucose metabolism in young overweight PCOS women might be a determinant of autonomic dysfunction [14]. Unfortunately, the cross sectional nature of the present study does not allow the evaluation of a causal association between autonomic dysfunction and IR and the elucidation of the mechanisms involved in the pathogenesis of both conditions.

It is known that inflammation plays an important role in the development and progression of atherosclerosis [39], and inflammatory markers such as CRP and WBCs count are strong predictors of cardiovascular events in healthy populations as well as patients with coronary heart disease [40].

Higher CRP levels and WBCs count have been described [4,7] in a wide PCOS women population suggesting an increased cardiovascular risk profile in these patients [2]. Observational studies showed a decreased autonomic nervous system activity related to inflammatory markers [22,23]. Recent experimental evidences suggest a role for the parasympathetic nervous system in the direct regulation of inflammation, pointing to the existence of a cholinergic anti-inflammatory reflex [24,25]. Recently, Vieira *et al. *[41] reported that post exercise HRR is independently associated with lower CRP in older sedentary individuals, suggesting an involvement of parasympathetic nervous system in regulating chronic inflammation in older adults. Moreover, it has been recently reported that the reduced cardiac adrenergic activity (evaluated by iodine-123-labeled metaiodobenzylguanidine uptake) observed in patients with glucose intolerance was associated to elevated pro-inflammatory cytokine levels [42].

The association between autonomic nervous system and systemic inflammation in young PCOS women after adjustment for common cardiovascular and metabolic confounders shed light on the complex mechanisms and the possible therapeutic strategies related to this endocrine-cardiometabolic disease.

Experimental and clinical evidences suggest that exercise training is an effective therapeutic intervention aimed at improving autonomic function as well as cardiopulmonary functional capacity [43-45]. In PCOS women, the exercise-induced improvement of cardiopulmonary and autonomic function should have been addressed to the improvement in insulin sensitivity, to the reduction in BMI, and to the powerful anti-inflammatory effect of training [46,47]. However, exercise training should be continued regularly in order to maintain the described beneficial effects [48].

Our previous prospective controlled data [46-48], in fact, confirmed that exercise training is effective in reducing BMI and improving insulin sensitivity markers in PCOS women, even thought no significant changes in sex hormones were observed. At this regard, it could be possible to hypothesize that the exercise induced improvement of autonomic function is mediated by BMI reduction and insulin sensitivity improvement.

Recently, Thompson *et al. *[49] studied the effect of weight loss on HRR in overweight and obese PCOS patients. After 10 weeks of diet, a significant improvement in HRR was observed in concert with a reduction in body weight, waist circumference, blood pressure, fasting insulin and glucose levels, HOMA score, T, FAI, and with an increase in SHBG [49]. In addition, HRR was significantly related to the reduction in body weight and waist circumference [49]. These findings demonstrated that weight loss can exert a beneficial role in reducing the cardiovascular risk in PCOS patients also improving the autonomic function [49].

Given the cross-sectional nature of the present study, it cannot be determined whether impaired autonomic function is the cause or effect of systemic inflammation. A potential limitation of our data is that we did not control for diet status, which may potentially confound the relation between HRR and inflammation markers. Because of the relation between cardiopulmonary functional capacity and HRR, strength of this study is the use of directly measured peak oxygen uptake as a covariate.

## Conclusion

In young PCOS women, abnormal HRR after exercise testing is significantly and closely associated to inflammatory markers, i.e. CRP and WBCs. These findings could suggest that in women with PCOS several alterations could act in concert contributing to increase cardiovascular risk profile of these patients.

## Competing interests

The authors declare that they have no competing interests.

## Authors' contributions

FG, FO, GL, AC, CV, MGT and SP conceived of the study, participated in its design and coordination and drafted the manuscript. FG performed the statistical analysis. All authors read and approved the final version of the manuscript.
